# Crowdfunding Campaigns for Paediatric Patients: A Cross-sectional Analysis of Success Determinants

**DOI:** 10.34763/jmotherandchild.20212503SI.d-21-00022

**Published:** 2022-04-30

**Authors:** Mikołaj Kamiński, Aleksandra Borys, Jan Nowak, Jarosław Walkowiak

**Affiliations:** 1 Department of Paediatric Gastroenterology and Metabolic Diseases, Poznan University of Medical Sciences, Poznan, Poland

**Keywords:** Crowdfunding, children, charity, Facebook, Cognitive Service, Poland

## Abstract

**Background:**

We aimed to identify factors of success in medical crowdfunding campaigns on the largest Polish platform: siepomaga.pl.

**Material and methods:**

All campaigns initialised by patients in the years 2009–2017 were included. The data comprised characteristics of the collections: financial target, raised sum, aim, type of disease, Facebook shares, age category, and the exact collection period. Campaign success was defined as collecting the target sum. Emotional expression on the main photograph was analysed using the Azure Cognitive Service. Logistic regression analysis was performed.

**Results:**

From a total of 2,656 collections, 1,725 (65%) were successful and 42.4 million EUR were raised in total. 2,024 (76.2%) of campaigns were dedicated to children. Successful collections not only received more donations, but were also supported, on average, with larger payments. Fortunate campaigns asked for less money and ended earlier (all p < 0.001). The odds of success were increased by: at least 50 Facebook shares (OR, 95% CI: 1.75, 1.46–2.10), the receiver being a child (1.46, 1.18–1.80), aim: dream come true (1.53, 1.06-2.20) or suffering from a congenital disease (1.34, 1.08–1.67), whereas financial target of no less than 4000 EUR (0.41, 0.34–0.52), aim: rehabilitation (0.51, 0.41-0.64), psychiatric disease (0.52, 0.38–0.71), and maladies of the eye or the ear (0.56, 0.39–0.81) were associated with campaign failure. After adjustment, dominance of happiness on the main photograph decreased the odds of success (0.71, 0.59–0.86).

**Conclusion:**

Younger age, lower financial goal, greater exposure on Facebook, aim, disease and emotional expression were associated with success of medical crowdfunding.

## Introduction

Crowdfunding, which enables financing through pooling of small contributions [1], is attractive and increasingly available to patients. In the era of social media, individual medical campaigns raise over one million euros (EUR) for specific needs, with successful collections breaking economic barriers and enabling access to the most sophisticated treatment modalities, which are not covered by health insurance [2]. This aspect may be crucial in countries with limited capacity for public healthcare but remains poorly explored in countries with a universal healthcare system, such as Poland. Polish individuals are obliged to pay a monthly public insurance fee, even if they have private health insurance. In Poland, underage individuals (below 18 years old) have guaranteed service in the public healthcare system regardless of parents’ insurance. Notwithstanding, public healthcare has limited capacity and treatment options; thus, families may seek help from other institutions. Up to 80% of Polish citizens use public healthcare, but only 36% of Poles declared that the current model of public health insurance is suitable for their needs [3]. Besides the governmental and private sector, nongovernmental organisations (NGOs) may also provide medical services, as well as financing them for those in need. Medical crowdfunding increases awareness of population health needs and engages the public in charity [4]. In this study, we focus on such crowdfunding collections, that is, for the needs of ill or disabled individuals.

Not all such campaigns meet their financial targets and it is unclear why [5]. It is possible that there is an emotional impact that conditions the viral spread and success of an appeal [1, 6, 7]. There are increasing concerns that highly emotive campaigns may outrival those that remain more reasonable but unspectacular [8]. Moreover, one of the biggest threats for crowdfunding, also dependent on cybersecurity and national regulations, is fraud [9, 10]. Therefore, the medical crowdfunding market should be vigilantly monitored, since it is a common good built on trust.

Moreover, understanding which causes garner support may provide insights into what kind of health needs are not met in universal healthcare systems [5]. However, collections for unproven or even risky therapies are not rare; these may hurt the beneficiaries and also induce others to follow [11, 12, 13]. It is not known if crowdfunders seek physicians’ advice regarding the choice of treatment. In any case, the topic is of interest to care providers, who might suggest that their patients undertake a crowdfunding campaign in order to support an evidence-based intervention.

Overall, the field is new and is intensively studied. Previous studies showed that the features of collections and a person in need might be related to crowdfunding campaign outcome [6, 8, 14, 15]. However, most of the studies were limited to a specific group of patients and did not undertake a broad survey. There is little known about what age groups, types of diseases, and aims are poised to benefit the most from crowdfunding campaigns.

Herein, we present detailed characteristics of collection for medical goals using the largest Polish platform as an example. We hypothesised that the features of the collections, such as patient age, financial target, aims, or diagnosis, were associated with the success of medical crowdfunding campaigns. The aim of the study was to identify factors of success in medical crowdfunding campaigns on the largest Polish platform: siepomaga.pl.

## Material and methods

### Data collection

Data from the medical crowdfunding webpage siepomaga. pl, managed by Siepomaga Foundation (Poznań, Poland) was analysed. The Foundation can manage crowdfunding campaigns due to general regulations concerning NGO activities in Poland. Siepomaga Foundation declared that the portal is the largest among Polish websites with collections for health needs. The Foundation charges 6% of the total amount raised in each collection. The Foundation reported authenticity of all crowdfunding campaigns on the website, and all campaigns initialised by individuals in the years 2009–2017 were included. The Foundation authenticates the crowdfunders based on scans of disclosed documentation. All campaigns for the needs of institutions or persons without a declared medical condition were excluded. The data was collected using Octoparse (Octopus Data Inc., CA, US) software dedicated for web scraping. Each record contained the substantial data of the collection: title of the campaign, aims, declared medical condition(s), total raised sum, reached percentage of the financial target, number of payments, name(s) of the receiver, number of shares of the campaign on Facebook, age of the recipient, place of residence (name of city/village and region) and URL of the main photograph. The Foundation agreed to the use of web scraping for the purpose of this study.

### Campaign characteristics

Data manipulation was conducted using *tidyr*, *stringr*, *qdapRegex*, *lubridate*, and *dplyr* packages of R [16]. All sums and payments were expressed in the local currency (PLN, Polish zloty). To convert PLN to EUR (euro), the currency rate was calculated as the mean of currency rates announced by the National Bank of Poland for the first and the last day of the collection. Successful collections were defined as those with the raised sum equal to or higher than the financial target of the collection. Each recipient was categorised as a child (younger than 18 years) or adult. Additionally, age subgroups were analysed: fetus – collection started before birth; newborn & infants < 1 year; toddlers, aged 1–3 years; preschool children, aged 4–5 years; school children, aged 6–12 years; adolescents, 13–17 years; adults, 18–65 years; seniors > 65 years. The following were also noted: recipient(s) sex, number (usually one only), and the place of residence, a city or village.

### Aims of the campaigns

The declared aims of the campaigns were categorised as costs of surgery, non-surgical treatment, rehabilitation, medical equipment, household needs and/or food, diagnostic investigation, follow-up costs, expenses related to a journey to a medical center, living in a nursing home, and making dreams come true. The latter category included nonmedical needs of the recipient, mostly related to entertainment, for example, buying a laptop or a ticket for the favorite world-class football team match. Each recipient could declare multiple aims of the collection.

### Medical conditions

Each declared medical condition was classified according to the International Statistical Classification of Diseases and Related Health Problems, 10^th^ Revision (ICD-10) [17]. We chose this classification due to its popularity and well-described criteria. In the case of a dubious or incomplete description of the declared medical condition, we carefully read the whole text of the campaign. When detailed information was lacking, we only assigned a broad ICD-10 category of the disease in question, taking note of all the campaigns with two or more declared conditions. Coexisting congenital heart defects were not counted separately.

Statistical analysis was performed with STATISTICA 12.0 (StatSoft, OK, US). Numerical data are presented as median (interquartile range [IQR]) and Boolean as number (percentage [%]). The Mann-Whitney U test and the chi-square test were used to compare successful vs. unsuccessful campaigns in Tables 1–3. The significance level was set at a p-value < 0.05. To assess factors that could influence the collection outcome (successful campaign, collection of 100% of financial target, was coded as one vs. unsuccessful campaign, collection of less than 100% of financial target, coded as null), univariate and multivariate logistic regression analyses were performed. For the multivariate logistic regression model, we selected all variables with a p-value below 0.1 in univariate regression analysis. We performed two additional multiple linear regression analyses. In the first model, the dependent was the collected sum, and in the second, it was the percentage of collection target raised.

**Table 1:** Comparison of financially successful vs. unsuccessful collections in years 2009–2017. Data presented as median (IQR)

Variable	All collections n = 2656 (100%)	Successful collections n = 1725 (65%)	Unsuccessful collections n = 931 (35%)	p-value
Target of the collection [EUR]	3,996 (1,464–14,474)	3,374 (1,138–13,326)	5,609 (2,396–16,534)	< 0.001
Total raised sum [EUR]	2,565 (927–9,325)	3,384 (1,154–13,561)	1,690 (775–4,629)	< 0.001
Raised target of the collection [%]	100 (53-100)	100 (100-101)	35 (18-57)	< 0.001
Total number of payments	311 (106–1,370)	371 (115–1,898)	244 (91–254)	< 0.001
Mean payment values [EUR]	7.87 (5.36–11.27)	8.50 (5.58–12.41)	6.93 (5.18–9.41)	< 0.001
Number of Facebook shares	48 (0–1,033)	86 (0–1,570)	7 (0–510)	< 0.001
Duration of the collection [days]	82 (26–167)	44 (14–105)	146 (91–254)	< 0.001

EUR – Euro

**Table 2:** Collection aims and recipient characteristics in years 2009–2017. Data presented as n (%)

Variable	All collections	Successful	Unsuccessful	p-value
Receiver lives in the city	1999 (75.3%)	1285 (74.5%)	714 (76.7%)	0.21
Sex: males	1406 (52.9%)	906 (52.5%)	500 (53.7%)	0.56
Collection for at least two children	821 (30.9%)	530 (30.7%)	291 (31.3%)	0.11
**Age: Child**	**2024 (76.2%)**	**1371 (79.5%)**	**653 (70.1%)**	**<0.001**
**Aim: Surgery**	**687 (25.9%)**	**477 (27.7%)**	**210 (22.6%)**	**0.004**
**Aim: Non-surgical therapy**	**619 (23.3%)**	**370 (21.4%)**	**249 (26.7%)**	**0.002**
**Aim: Rehabilitation**	**619 (23.3%)**	**331 (19.2%)**	**288 (30.9%)**	**<0.001**
Aim: Medical equipment	523 (19.7%)	350 (20.3%)	173 (18.6%)	0.29
**Aim: ‘Dream come true’**	**294 (11.1%)**	**241 (14.0%)**	**53 (5.7%)**	**<0.001**
Aim: Household needs and/or food	74 (2.8%)	46 (2.7%)	28 (3.0%)	0.61
Aim: Diagnostic investigations	25 (0.9%)	20 (1.2%)	5 (0.5%)	0.11
Aim: Follow-ups costs	11 (0.4%)	6 (0.3%)	5 (0.5%)	0.47
Aim: Cost of a journey to a medical center	7 (0.3%)	4 (0.2%)	3 (0.3%)	0.66
Aim: Costs of living in a nursing home	7 (0.3%)	5 (0.3%)	2 (0.2%)	0.72

**Table 3:** Diseases motivating collections in years 2009–2017. Data presented as n (%)

Variable	All collections	Successful	Unsuccessful	p-value
At least two different medical conditions declared	821 (30.9%)	530 (30.7%)	291 (31.3%)	0.78
Infectious diseases (ICD-10 A00-B99) n(%)	12 (0.5%)	9 (0.5%)	3 (0.3%)	0.46
Neoplasm (ICD10 C00-D49) n(%)	523 (19.7%)	355 (20.6%)	168 (18.0%)	0.12
Diseases of the blood (ICD10 D50-D89) n(%)	51 (1.9%)	34 (2.0%)	17 (1.8%)	0.79
Endocrine, nutritional and metabolic diseases (ICD10 E00-E89) n(%)	183 (6.9%)	115 (6.7%)	68 (7.3%)	0.54
**Psychiatric diseases (ICD10 F01-F99) n(%)**	**206 (7.8%)**	**107 (6.2%)**	**99 (10.6%)**	**<0.001**
Diseases of the nervous system (ICD10 G00-G99) n(%)	763 (28.7%)	476 (27.6%)	287 (30.8%)	0.08
**Eye or ear diseases (ICD10 H00-H59) n(%)**	**134 (5.0%)**	**73 (4.2%)**	**61 (6.6%)**	**0.01**
Cardiovascular diseases (ICD10 I00-I99) n(%)	86 (3.2%)	56 (3.2%)	30 (3.2%)	0.97
Respiratory diseases (ICD10 J00-J99) n(%)	34 (1.3%)	22 (1.3%)	12 (1.3%)	0.98
Digestive diseases (ICD10 K00-K95) n(%)	45 (1.7%)	30 (1.7%)	15 (1.6%)	0.81
Dermatological diseases (ICD10 L00-L99) n(%)	16 (0.6%)	10 (0.6%)	6 (0.6%)	0.84
Muscuoskeletal and connective tissue diseases (ICD10 M00-M99) n(%)	54 (2.0%)	34 (2.0%)	20 (2.1%)	0.76
Genitourinary diseases (ICD10 N00-N99) n(%)	18 (0.7%)	12 (0.7%)	6 (0.6%)	0.88
Pregnancy or perinatal complications (ICD 10 O00-P96) n(%)	182 (6.9%)	109 (6.3%)	73 (7.8%)	0.14
**Congenital diseases (ICD10 Q00-Q99) n(%)**	**888 (33.4%)**	**623 (36.1%)**	**265 (28.5%)**	**<0.001**
**Injuries (ICD10 S00-T88) n(%)**	**68 (2.6%)**	**34 (2.0%)**	**34 (3.7%)**	**0.01**

Details on emotional expression analysis and statistical methods are described in Supplementary File 1.

## Results

A total of 2,859 campaigns (2009–2017) were screened, and 2,656 collections met the inclusion criteria. Of these, 1,725 (65%) were successfully finished. In the period considered, the number of campaigns per year (Supplementary File 2), as well as financial targets, raised sums (Supplementary File 3), and the number of campaign shares on Facebook (Supplementary File 4) increased. The sum of all raised funds amounted to 42.4 million EUR. The ten most successful campaigns collectively raised almost 9 million EUR (Supplementary File 3). The most prevalent medical conditions are presented in Supplementary File 6.

### Factors of success

Successful campaigns were compared with those that did not reach their goals (Tables 1–3). The successful campaigns raised higher sums and received more payments, which were greater (Table 1). Success was associated with more Facebook shares, lower financial targets, and shorter durations (Table 1). Moreover, a larger share of successful recipients were children and patients suffering from congenital disorders (Tables 2 and 3). Unsuccessful collections more frequently concerned psychiatric illness, eye and ear disease, and injuries (Table 3).

For the univariate logistic regression analysis, rounded medians of the financial target of the collection were used (n = 3,996 ≈ 4,000 [EUR]) and the number of shares on Facebook (n = 48 ≈ 50 [n]). The results of univariate logistic regression analysis are presented in Supplementary File 7.

**Figure 1:**
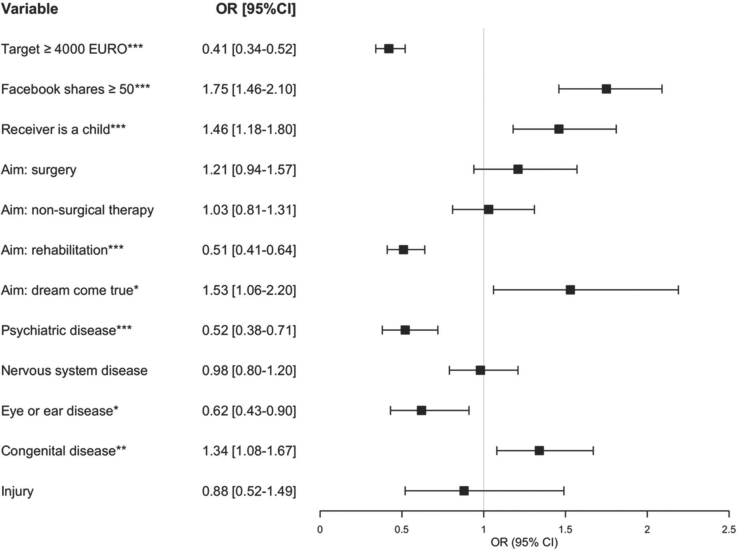
Multivariate regression analysis of all campaigns (n = 2656). Dependent variable: success of the campaign. *p < 0.05; **p < 0.01; ***p <0.001. EUR - Euro, CI - Confidence interval, OR - Odds ratio.

In multivariate logistic regression analysis, the odds of success were increased by the number of shares on Facebook ≥ 50 (OR = 1.75; 95% CI: 1.46–2.10), receiver of funds being a child (OR = 1.46; 95% CI: 1.18–1.80), aim: dream come true (OR = 1.53; 95% CI: 1.06-2.20) or suffering from congenital disease (OR = 1.34; 95% CI: 1.08–1.67), while collection target ≥ 4000 EURO (OR = 0.41; 95% CI: 0.34–0.52), aim: rehabilitation (OR = 0.51; 95% CI: 0.41-0.64) receiver suffering from psychiatric disease (OR = 0.52; 95% CI: 0.38–0.71), or eye or ear disease (OR = 0.62; 95% CI: 0.43–0.90) were independently associated with poorer campaign outcome (Fig. 1).

We compared the chances of the collection success of each age subcategory (Supplementary File 8). The newborn and infants (χ^2^ (1, n = 2656) = 10.28; p < 0.001) and toddlers (χ^2^ (1, n = 2656) = 51.73; p < 0.001) had significantly higher chance of success, while school-aged (χ^2^ (1, n = 2656) = 3.94; p = 0.047) and adults (χ^2^ (1, n = 2656) = 31.64; p < 0.001) had significantly lower chance of success.

### Emotion analysis

The face emotion analysis was conducted for n = 2129 main photographs. The dominant emotions presented on the images were as follows [n(%)]: neutral 1046 (49.1%), happiness 1007 (47.3%), surprise 40 (1.9%), sadness 31 (1.5%), contempt 3 (0.1%) and anger 2 (0.1%). The median sentiment of n = 2129 campaigns titles was equal to 0.51 (0.45–0.57). The results of univariate logistic regression analysis are presented in Supplementary File 9.

In multivariate logistic regression analysis, the odds of success were increased by number of shares on Facebook ≥ 50 (OR = 1.66; 95% CI: 1.36–2.03), the recipient of the funds being a child (OR = 1.32; 95% CI: 1.05–1.66) or suffering from congenital disease (OR = 1.34; 95% CI: 1.08–1.66), while the collection target ≥ 4000 EUR (OR = 0.37; 95 %CI: 0.29–0.47), aim: rehabilitation (OR = 0.46; 95% CI: 0.36-0.60), psychiatric illness (OR = 0.54; 95% CI: 0.38–0.76) or dominant face emotion on the main campaign photograph being happiness (OR = 0.71; 95% CI: 0.59–0.86) were independently associated with a poorer campaign outcome (Supplementary File 10).

In multiple linear regression analysis, Facebook shares, the receiver being a child, and spending funds on surgery or nonsurgical therapy were associated with greater total raised funds (adjusted R^2^=0.34, F(4,2651)=342.3, p < 0.001) (Supplementary File11a). Moreover, the raised percentage of campaign target increased with Facebook shares, the receiver being a child, and spending funds to make a dream come true. On the other hand, it decreased when the campaigner declared spending funds on rehabilitation and/or psychiatric, eye or ear disease (adjusted R^2^=0.05, F(6, 2649)=23.61, p < 0.001) (Supplementary File 11b).

## Discussion

This study, which analysed the largest number of medical crowdfunding collections coming from Europe to date, revealed patterns associated with campaign success. The five factors linked to goal attainment were: a modest financial target, a lower patient age, a greater number of shares on Facebook, and a specific category of the receiver’s malady.

Comprehensive characteristics of each campaign were obtained, including the patient, the aim, the outcome, and the emotional content. This example illustrates the increase in medical crowdfunding in Poland, explores the range of collection goals, financial targets, also the link to the world of social media. The results showed that medical crowdfunding was used to raise funds for a wide spectrum of unmet health-related needs of people from unborn fetuses to seniors. Therefore, medical crowdfunding provides equality of opportunity. Moreover, the medical crowdfunding campaigns may indicate which diseases and treatments may require additional public health funds and which people may need further social assistance. However, due to the limited resources of the donors, the outcomes of campaigns are diverse.

### Financial target

Negative association between the collection’s financial target and its outcome contrasts with previous research. A higher financial target increased the odds of success in a study by Durant et al., with each 1000 USD raising the chance of success by 0.6% [6]. Similar results were reported for prostate and breast cancer treatment [15] as well as orthopedic surgery [14]. However, those studies investigated campaigns with a narrow group of goals, while we included all types of medical goals.

It should be stressed, however, that on many platforms, including GoFundMe and siepomaga.pl, campaign success is not required to receive the collected funds. As much as 40% of GoFundMe campaigns fall short of their targets, leaving crowdfunders with partial but often considerable support [8]. In this analysis, the rate of failure was lower. We hypothesise that this moderate difference could be linked to a relatively higher number, and greater generosity and activity of the donors’ community, compared with the number of campaigns, as well as the lower financial targets in Poland. Moreover, crowdfunding in North America has a longer history than in Poland, and it may be associated with the limited trust of potential donors. We described the first 8 years of the largest crowdfunding portal in Poland, during which some campaigners might have overestimated donor generosity: unsuccessful campaigns had higher financial targets compared with successful campaigns. The median target of the collection was equal to 4000 EUR, while the median and average of monthly net salary in Poland in the years 2009–2017 ranged respectively between 500 to 600 and 700 to 800 EUR [18].To date, the analysis of medical crowdfunding in the Western world included richer English-speaking countries such as Canada, the United Kingdom, and the United States, and Ireland as well as Germany [12, 14, 15, 19, 20, 21].

It is important to note that about 75% of the unsuccessful collections raised less than 60% of the financial target. The donors were more eager to support the campaigns approaching the financial goal, which may be explained by the social proof phenomenon. The social proof is a subjective assumption that the surrounding people possess more knowledge about the current situation and make the right decisions [22]. The collections with a lower financial target may quickly achieve a higher percentage of the raised sum; consequently, the social proof threshold is crossed. However, this speculation requires further investigation.

### Social media exposure

The pivotal role of social media in modern crowdfunding was initially described in other contexts [23, 24]. In medicine, the value of social capital was confirmed by Paulus et al., who explored 105 GoFundMe campaigns [7]. In another study, which considered crowdfunding for whole exome genetic sequencing (n=11), 65% of the receiver’s social media posts were shared, and the click rate for embedded links was 70% [4]. Moore raised the concern that collections of patients without social media networks may be marginalised [25]. In our view, this is a consequence of the amplification of pre-existing social mechanisms, which already influenced crowdfunding before the advent of Web 2.0.

In this study, a higher number of shares on Facebook was beneficial. However, at least a quarter of successful collections did not have any Facebook shares at all and donations likely came from family, friends, and webpage visitors. Further research could focus on factors that determine whether a campaign gains viral momentum. It is possible that not only the number of shares is important, but also the social impact of the person who shares. This could prove to be a powerful factor, as in this analysis one Facebook share could be converted to ~ 6 EUR.

### Disease category

Potential donors might intuitively discern serious but amenable conditions. In this dataset, the odds of success were dependent on the diagnosis. We observed that success was more probable with congenital disease, contrary to the presence of, for instance, psychiatric disease or eye or ear illness. The most frequent congenital malformations were heart defects, which were also associated with a better collection outcome, whereas poorer results were achieved with the most prevalent of the sample’s psychiatric diagnoses, autism spectrum disorders. A related point to consider is that psychiatric illness may be stigmatising; for example, opiate-addicted Canadians raised only 1.6% of the amount requested through GoFundMe and YouCaring [26]. Basing on the obtained results, we speculate that donors were more eager to support patients with conditions perceived as curable. Moreover, the collections for children’s needs are more likely to achieve financial success relative to those which concern adults [27].

### Campaign goals

The influence of diagnosis may potentially be confounded by the aim of a campaign, such as higher chances of success for surgical treatment or lower for rehabilitation. It is yet another factor that adds to the picture of a successful campaign, the knowledge of which may be useful for clinicians wishing to empower their patients by crowdfunding.

The analysis of the most frequent aims should provide knowledge about gaps in public health insurance coverage. For example, in another Canadian study, the aims of the collections reflected the insufficiencies of public cancer care [28]. In this study, 22% of collections raised money for rehabilitation and 19% for medical equipment, which may mirror an insufficient coverage of these needs in Poland [29]. Indeed, the share of individuals with longstanding limitations due to health problems is higher in Poland than is typical in the European Union [30].

The data reveal that a declaration of spending funds for rehabilitation decreased the odds of campaign success. Rehabilitation may be perceived as chronic management of the incurable condition, and for this reason, donors might have been less eager to support such collections. It might also be hypothesised that the rehabilitation is less attractive for donors because it lacks the appeal of novel therapeutic methods. It is possible that rehabilitation could be perceived as more worthy of support when presented as yielding tangible results: durably transformative for the lifestyle and a milestone on the way to achieving self-sufficiency.

Collections for entertainment purposes are controversial. In this work, as many as 11% of collections proposed ‘to make a dream come true’. Despite the controversy this goal raises, donors were willing to support it. This may reflect the preoccupation of the public with the quality of life of the chronically or terminally ill. It is also possible that those campaigns were successful owing to a combination of young patient age, low financial targets, and highly emotional context. This finding also reminds us that many donors feel that leading a meaningful life may be more important than providing advanced medical care. The investigation of this phenomenon requires further study.

### Emotion analysis

Campaign-related photographs may elicit a range of emotions, including compassion, awkwardness, even guilt. Strikingly, in this study, happiness was associated with a poor outcome. It seems plausible that ostensibly happier patients were judged not to require aid. Loeb et al. found that collections exhibiting more photographs with family members were more likely to raise the target sum than those with medical-themed photographs [15]. It is unclear whether vivid images make donors more susceptible to aid others or lead to desensitisation. The title sentiment did not associate with collection success. In the study by Durand et al., a more positive emotional campaign content related to the achievement of organ transplant collection targets [6]. Berliner et al. found that campaigns with longer descriptions were more likely to achieve financial success, a factor that we did not explore [8]. The pressure to provide convincing content to fuel the campaign may pose a risk to a patient’s privacy. Emotional communication is at the very core of medical crowdfunding, and it may be expected to be highly culture-dependent.

### Limitations

This work was limited by the lack of information on the share and payment distribution over time, which could reveal the dynamics of crowdfunding and direct relationships with social media. The description and information about the crowdfunder are set before the beginning of the collection. Therefore, the characteristics may play a causal role for the final success. The presented study is retrospective and observational, and thus, we may only indicate correlations but cannot infer cause-effect relationships. These newly identified associations may be verified by further prospective studies or behavioral economic experiments. Furthermore, neither the length of descriptions nor the content of the illustrations were analysed. Face API failed to analyse 20% of the photographs. The presented multivariate models are complex, thus the results should be interpreted with caution. The assignment of ICD-10 codes was based on the given diagnosis, so it might be inaccurate and/or lacking comorbidities. Last but not least, this study adds value to the literature of medical crowdfunding but the observation may be related to local economic, social, and cultural settings; the outcomes cannot be extrapolated for the whole world. However, they can be generalised to Poland and Central/Eastern Europe, where little research has been done on the topic thus far.

## Conclusion

In general, the analysed medical crowdfunding campaigns had high chances of success. The odds are further increased by the patient’s young age, lower financial target, greater exposure on Facebook, aims, specific diagnoses, and emotional content. Our findings confirm previously described ethical concerns that medical crowdfunding success may depend on the campaign features that are not limited to the severity of health problems.

## Key points

- Medical crowdfunding has become a global phenomenon, which remains under-researched.
- What are factors of success in medical crowdfunding campaigns on the largest Polish platform, siepomaga.pl?
- We analysed patients’ needs and the factors of success in almost a decade of medical crowdfunding data from the largest Polish website dedicated to this cause.
- In the era of social media, individual medical campaigns may break economic barriers and enable access to the most sophisticated treatment modalities. Not all crowdfunding campaigns meet their financial targets.
- Success of the collection is associated with young age, social media shares, aims, disease type, and emotions.
- Medical crowdfunding heralds a change in healthcare as citizens directly choose which needs deserve coverage.
