# Supplementary material for: Crowdfunding Campaigns for Paediatric Patients: A Cross-sectional Analysis of Success Determinants
Source: J Mother Child. 2022 Apr 30;25(3):209–17. doi: 10.34763/jmotherandchild.20212503SI.d-21-00022 (PMC9097654; doi:10.34763/jmotherandchild.20212503SI.d-21-00022)
Supplement: Supplementary file 1 [file jmotherandchild-25-209_sm.pdf]

Supplementary File 1

Material and methods appendix

Emotional expression analysis

Additional analyses were performed to estimate the emotional factors of the collection using tools from Azure Cognitive Services platform (Microsoft Corporation, WA, US): Face API and Text Analytics API. Since a number of the campaign websites on Facebook present both the collection title and the main photo, those features were analysed. The Azure Face API detects and analyses human faces, providing information on emotions in a given image: happiness, anger, contempt, fear, sadness, surprise or a neutral expression. The outcome is expressed as a percentage of each emotion in the analysed image. Al-Omair reported that The Azure Face API had the highest accuracy among investigated recognition services, with average accuracy for happiness equal to 100% [1]. Face emotion analysis was obtained from collected URLs of the main campaign photograph and assigned the detected dominant emotion to each record. If the Face API does not recognise the human face, it does not generate emotion parameters. In the case of multiple human faces, we calculated the average emotional percentage. Azure Text Analytics is also a machine learning algorithm, which was used to identify emotional expressions, also called sentiments, from the campaign titles in the Polish language. The sentiment is represented by a number ranging from 0 to 1, with 0 signifying a completely negative emotional content and 1 a most positive one.

Statistical analysis

Statistical analysis was performed with STATISTICA 12.0 (StatSoft, OK, US). The normality of variable distribution was tested using the Kolmogorov-Smirnov test with the Lilliefors correction. Due to a lack of normality, non-parametrical tests were conducted. Numerical data are presented as median (interquartile range [IQR]) and Boolean as number (percentage [%]). The Mann-Whitney U test and the chi-square test were used to compare successful vs. unsuccessful campaigns in Tables 1–3.

To assess factors that could influence the collection outcome (successful campaign, collection of 100% of financial target – coded as one vs. unsuccessful campaign, collection of less than 100% of financial target – coded as null), univariate and multivariate logistic regression analyses were performed. In the univariate logistic regression model, the independent factors were: financial target of the collection equal or above the rounded median, number of shares on Facebook equal or above the rounded median, place of living of the receiver (city counted as 1, village as 0), sex of the recipient (at least one male receiver), collection for at least two children, receiver being a child (declared age < 18 years old), aims of the collection, at least two different medical conditions, medical condition.

For the multivariate logistic regression model, we selected all variables with a p-value below 0.1 in univariate regression analysis. Because Azure Cognitive Service did not perform face emotions analysis for all campaigns, additional logistic regression analyses were performed for the subset with Face API results. The extra independent variables were dominant face emotion on the main campaign photo: happiness and the sentiment of the collection title. The significance level was set at a p-value < 0.05. Figures were generated using the *ggplot2, ggthemes, forestplot* and *radarchart* packages of R [2]. We performed two additional multiple linear regression analyses. In the first model, the dependent was the collected sum, and in the second, it was the percentage of collection target raised. To the initial models, we assigned variables with p-value below 0.1 in a univariate linear regression model. From the secondary models, we excluded all independent variables with p-value equal or above 0.05 in the multiple linear regression model.

[1] Al-Omair OM, Huang S. A Comparative Study on Detection Accuracy of Cloud-Based Emotion Recognition Services. In: Proceedings of the 2018 International Conference on Signal Processing and Machine Learning - SPML '18. Shanghai, China:ACMPress2018.142–8.doi:10.1145/3297067.3297079

[2] Wickham H. ggplot2: elegant graphics for data analysis. Second edition. Cham: Springer 2016.

Supplementary File 2

Total number of collections initialized in 2009 to 2017

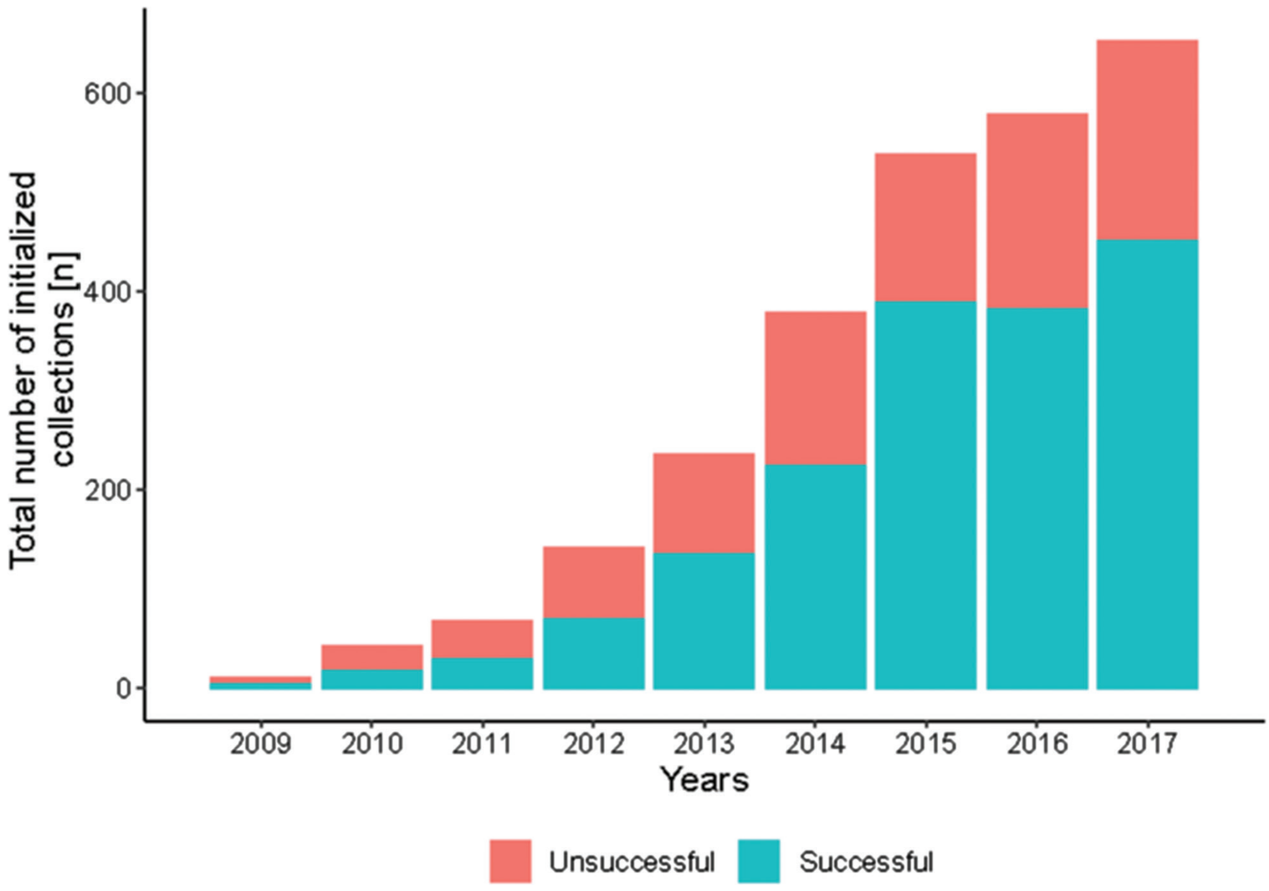

Figure S2. Financial targets and the sums raised by collections in 2009–2017.

Supplementary File 3

Financial targets and the sums raised by collections in 2009–2017.

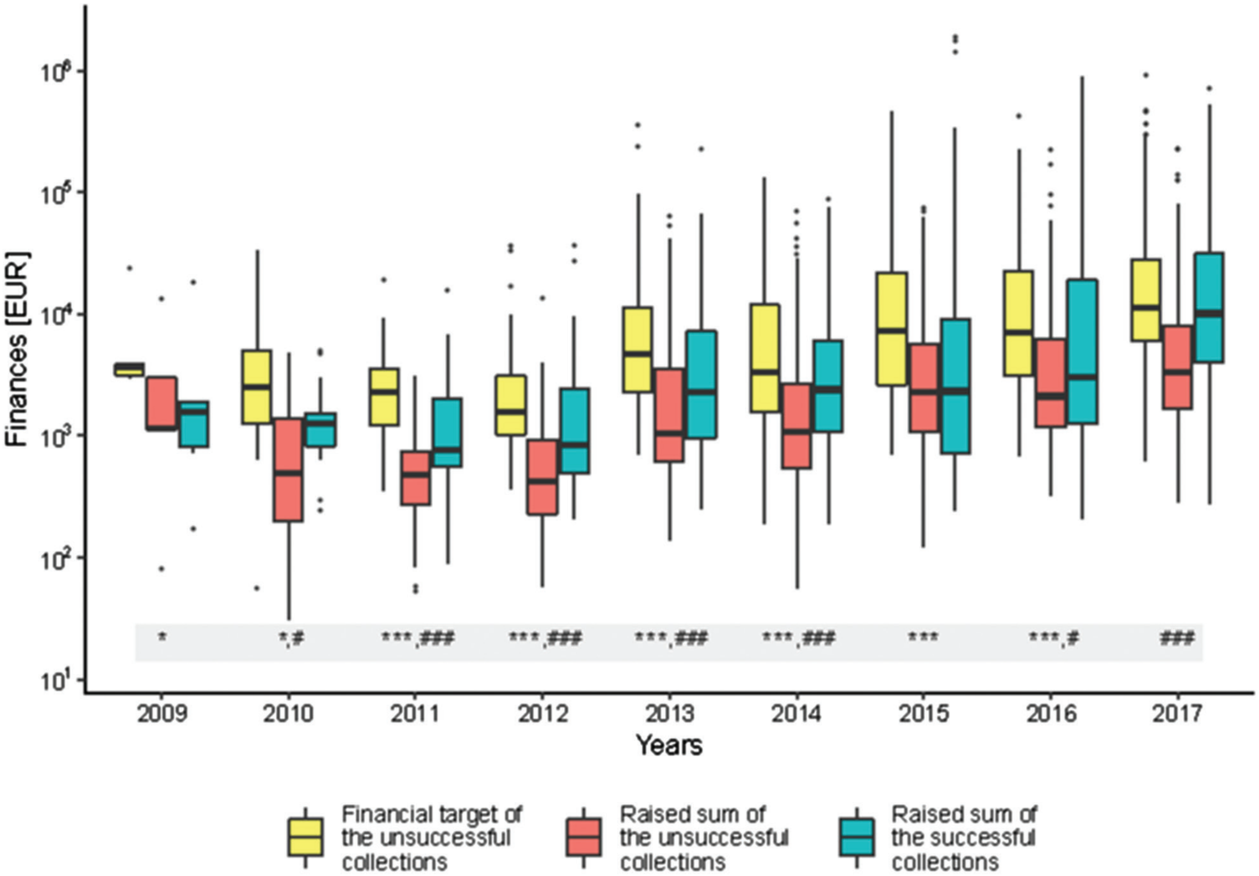

Figure S3. Comparison of the financial targets of unsuccessful collections and sums raised by successful collections: *p < 0.05; **p < 0.01; ***p <0.001.
Comparison between sums raised by unsuccessful and successful collections: #p < 0.05; ##p < 0.01; ###p <0.001.
EUR - Euro.

Supplementary File 4

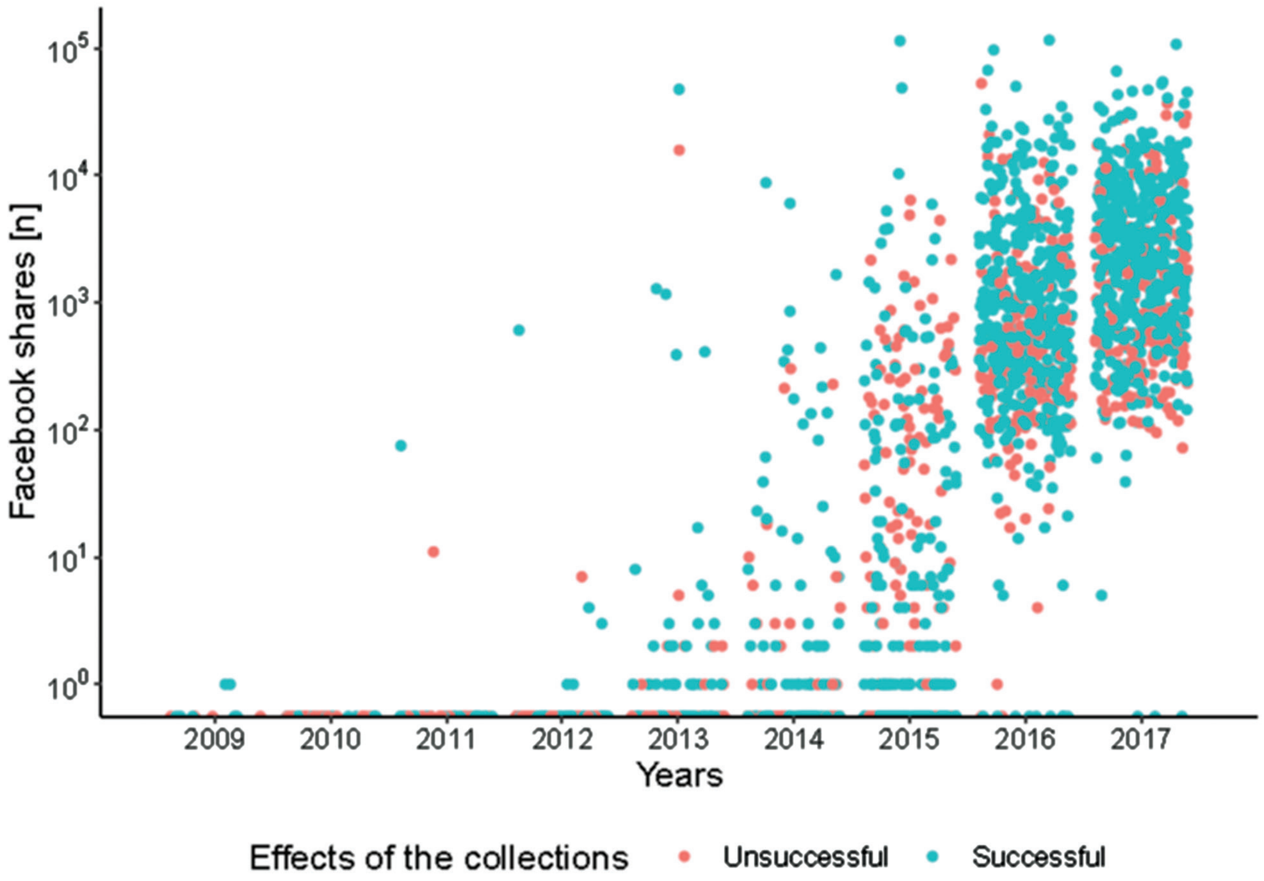

Figure S4. The number of Facebook shares in 2009 to 2017.

Supplementary File 5

Top ten successful collections on the crowdfunding website (www.siepomaga.pl)

Diagnosis	Aim	Age group	Collected sum (EUR)	URL
Epidermolysis bullosa	Allo-HSCT and stem cells transplantation, Minnesota, US	Infant	1,907,876	https://www.siepomaga.pl/kajtek
Tetralogy of Fallot with pulmonary atresia	Cardiac surgery, Stanford, US	School age	1,769,558	https://www.siepomaga.pl/emil
Epidermolysis bullosa	Allo-HSCT and stem cells transplantation, Minnesota, US	School age	1,434,060	https://www.siepomaga.pl/nomorepain
Progressive dystonia, VAC14 mutation	Stem cells treatment, US	School age	900,467	https://www.siepomaga.pl/kordian
Tetralogy of Fallot with pulmonary atresia	Cardiac surgery, Stanford, US	Adolescent	721,594	https://www.siepomaga.pl/sercejulii
Choroid plexus carcinoma, Li-Fraumeni syndrome	Treatment in Nationwide Children's Hospital, US	Preschool	519,479	https://www.siepomaga.pl/ocalic-jedrusia
Choroid plexus carcinoma, Li-Fraumeni syndrome	Treatment in Nationwide Children's Hospital, US	School age	497,349	https://www.siepomaga.pl/ocalic-tymka
Neuroblastoma, stage IV	Immunotherapy in Greifswald, Germany	Toddler	456,598	https://www.siepomaga.pl/filip-banas
Neuroblastoma, stage IV	Immunotherapy in Greifswald, Germany	School age	367,426	https://www.siepomaga.pl/ameliaszewczyk
Relapsed acute lymphoblastic leukemia, B-cell	Blinatumomab	Adult	341,239	https://www.siepomaga.pl/glinko

Allo- HSCT – Allogenic Hematopoietic stem cell transplantation; EUR – Euro; URL – Uniform Resource Locator; US – United States.

Supplementary File 6

Top 15 most prevalent medical conditions on the crowdfunding website (www.siepomaga.pl) in 2009–2017

Diagnosis (ICD-10)	Number of cases	Number of successful collections (success rate) [n(%)]
All	2565	1725 (65%)
**Congenital heart defects (Q20-Q24)**	**415**	**316 (76%)*** **
**Cerebral palsy (G80)**	**335**	**189 (56%)*** **
Epilepsy (G40)	197	117 (59%)
**Central nervous system tumour (C71)**	**125**	**97 (78%)** **
**Autism spectrum disorders (F84)**	**123**	**58 (47%)*** **
Cystic fibrosis (E84)	96	60 (63%)
Paraplegia and quadriplegia (G82)	92	51 (55%)
**Leukemia (C91)**	**89**	**87 (80%)** **
Spina bifida (Q05)	87	52 (60%)
Congenital hydrocephalus (Q03)	87	56 (64%)
**Spinal muscular atrophy and related syndromes (G12)**	**81**	**62 (77%)***
Lack of expected normal physiological development in childhood and adults (R62)	60	32 (53%)
Other congenital malformations of brain (Q04)	58	37 (64%)
Deafness (H90)	53	28 (53%)
Preterm labor (O60)	52	35 (67%)

Statistical significance was marked as follows: *p<0.05; **p<0.01; ***p<0.001 (Pearson's chi-square test).

Supplementary File 7

Univariate regression analysis. Dependent variable: financial success of the collection.

Variable	OR (95% CI)	p-value	Variable	OR (95% CI)	p-value
**Target of the collection ≥ 4,000 [EUR]**	**0.61 (0.52-0.72)**	**<0.001**	Infectious disease (ICD-10 A00-B99) n(%)	1.62 (0.44-6.01)	0.47
**Number of shares on Facebook ≥ 50 [n]**	**1.20 (1.02-1.41)**	**0.02**	Neoplasm (ICD10 C00-D49) n(%)	1.18 (0.96-1.44)	0.12
Receiver lives in the city n(%)	0.89 (0.74-1.07)	0.21	Disease of the blood (ICD10 D50-D89) n(%)	1.08 (0.60-1.94)	0.80
Sex: males n(%)	0.95 (0.81-1.12)	0.56	Endocrine, nutritional and metabolic disease (ICD10 E00-E89) n(%)	0.91 (0.66-1.24)	0.54
Collection for at least two children n(%)	0.65 (0.37-1.12)	0.12	**Psychiatric disease (ICD10 F01-F99) n(%)**	**0.56 (0.42-0.74)**	**<0.001**
**Age: children n(%)**	**1.65 (1.37-1.98)**	**<0.001**	**Disease of the nervous system (ICD10 G00-G99) n(%)**	**0.86 (0.72-1.02)**	**0.08**
**Aim: Surgery n(%)**	**1.31 (1.09-1.58)**	**<0.01**	**Eye or ear disease (ICD10 H00-H59) n(%)**	**0.63 (0.44-0.89)**	**0.01**
**Aim: Non-surgical therapy n(%)**	**0.75 (0.62-0.90)**	**<0.01**	Cardiovascular disease (ICD10 I00-I99) n(%)	1.01 (0.64-1.58)	0.97
**Aim: Rehabilitation n(%)**	**0.53 (0.44-0.64)**	**<0.001**	Respiratory disease (ICD10 J00-J99) n(%)	0.99 (0.49-2.01)	0.98
Aim: Medical equipment n(%)	1.12 (0.91-1.37)	0.29	Digestive disease (ICD10 K00-K95) n(%)	1.08 (0.58-2.02)	0.81
**Aim: "Dream come true" n(%)**	**2.69 (1.97-3.66)**	**<0.001**	Dermatological disease (ICD10 L00-L99) n(%)	0.90 (0.33-2.48)	0.84
Aim: Household needs and/or food n(%)	0.88 (0.55-1.42)	0.61	Muscuoskeletal and connective tissue disease (ICD10 M00-M99) n(%)	0.92 (0.52-1.60)	0.76
Aim: Diagnostic investigations n(%)	2.17 (0.81-5.81)	0.12	Genitourinary disease (ICD10 N00-N99) n(%)	1.08 (0.40-2.89)	0.88
Aim: Follow-ups costs n(%)	0.65 (0.20-2.12)	0.47	Pregnancy or perinatal complication (ICD 10 O00-P96)n(%)	0.79 (0.58-1.08)	0.14
Aim: Cost of a journey to a medical center n(%)	0.72 (0.16-3.22)	0.67	**Congenital disease (ICD10 Q00-Q99) n(%)**	**1.42 (1.20-1.69)**	**<0.001**
Aim: Costs of living in a nursing home n(%)	1.35 (0.26-6.97)	0.72	**Injury (ICD10 S00-T88) n(%)**	**0.53 (0.33-0.86)**	**0.01**
At least two different medical conditions declared n(%)	0.98 (0.82-1.16)	0.78			

CI – Confidence Interval; EUR – Euro; OR – Odds Ratio.
Variables with p-value <0.1 were bolded.

Supplementary File 8

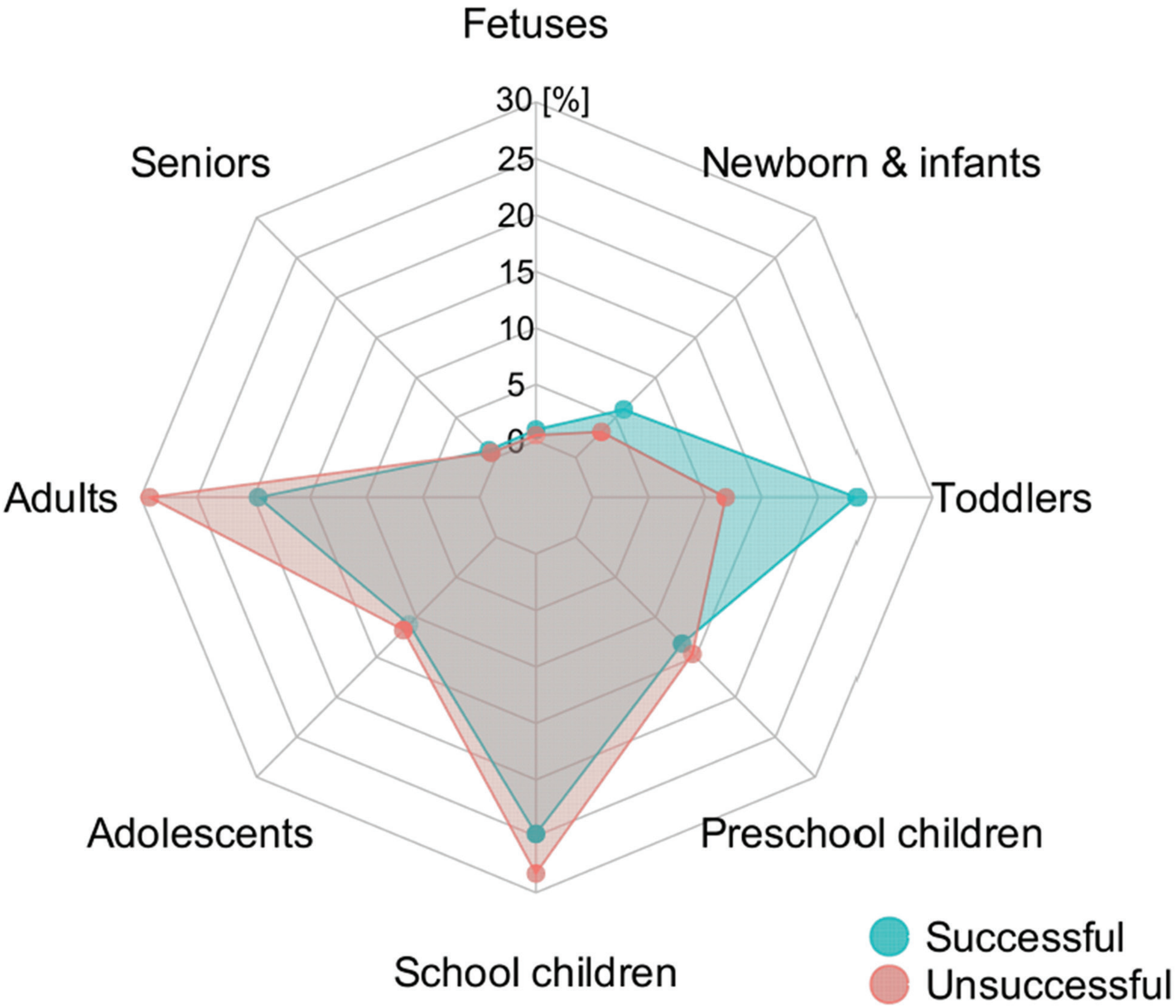

Figure S8. Age categories of the recipient in successful and unsuccessful collections Fetus, collection started before birth; newborn & infants < 1 year; toddlers, 1–3 years; preschool children, 4–5 years; school children, 6–12 years; adolescents, 13–17 years; adults, 18–65 years; seniors > 65 years.

Supplementary File 9

Univariate regression analysis. Dependent variable: financial success of the collection. N=2129.

Variable	OR (95% CI)	p-value	Variable	OR (95% CI)	p-value
**Target of the collection ≥ 4,000 [EUR]**	**0.55 (0.46-0.66)**	**<0.001**	Neoplasm (ICD10 C00-D49) n(%)	1..12 (0.90-1.39)	0.32
**Number of shares on Face-book ≥ 50 [n]**	**1.16 (0.97-1.38)**	**0.09**	Disease of the blood (ICD10 D50-D89) n(%)	1.69 (0.82-3.50)	0.16
Receiver lives in the city n(%)	0.85 (0.69-1.05)	0.12	Endocrine, nutritional and metabolic diseases (ICD10 E00-E89) n(%)	0.97 (0.70-1.37)	0.88
Sex: males n(%)	0.94 (0.79-1.12)	0.47	**Psychiatric disease (ICD10 F01-F99) n(%)**	**0.59 (0.43-0.81)**	**0.001**
Collection for at least two children n(%)	0.78 (0.43-1.43)	0.43	Disease of the nervous system (ICD10 G00-G99) n(%)	0.88 (0.72-1.07)	0.19
**Children n(%)**	**1.55 (1.27-1.90)**	**<0.001**	**Eye or ear disease (ICD10 H00-H59) n(%)**	**0.70 (0.48-1.03)**	**0.07**
**Aim: Surgery n(%)**	**1.22 (0.99-1.50)**	**0.06**	Cardiovascular disease (ICD10 I00-I99) n(%)	1.24 (0.73-2.10)	0.43
**Aim: Non-surgical therapy n(%)**	**0.74 (0.60-0.91)**	**<0.01**	Respiratory disease (ICD10 J00-J99) n(%)	1.35 (0.59-3.13)	0.48
**Aim: Rehabilitation n(%)**	**0.50 (0.41-0.62)**	**<0.001**	Digestive disease (ICD10 K00-K95) n(%)	1.47 (0.68-3.22)	0.33
Aim: Medical equipment n(%)	1.15 (0.92-1.44)	0.21	Dermatological disease (ICD10 L00-L99) n(%)	1.08 (0.36-3.23)	0.89
**Aim: “Dream come true” n(%)**	**2.72 (1.97-3.76)**	**<0.001**	Muscuoskeletal and connective tissue disease (ICD10 M00-M99) n(%)	0.84 (0.45-1.58)	0.59
Aim: Household needs and/or food n(%)	1.09 (0.64-1.85)	0.74	Genitourinary disease (ICD10 N00-N99) n(%)	1.08 (0.36-3.23)	0.89
Aim: Diagnostic investigations n(%)	1.81 (0.58-5.62)	0.31	**Pregnancy or perinatal complication (ICD 10 O00-P96) n(%)**	**0.72 (0.51-1.02)**	**0.07**
Aim: Follow-ups costs n(%)	0.60 (0.17-2.07)	0.42	**Congenital disease (ICD10 Q00-Q99) n(%)**	**1.37 (1.13-1.66)**	**0.001**
Aim: Cost of a journey to a medical center n(%)	1.20 (0.22-6.56)	0.83	**Injury(ICD10 S00-T88) n(%)**	**0.44 (0.25-0.75)**	**0.003**
Aim: Costs of living in a nursing home n(%)	2.40 (0.27-21.53)	0.43	**Dominant face emotion: happiness n(%)**	**0.78 (0.65-0.93)**	**0.005**
At least two different medical conditions declared n(%)	0.96 (0.79-1.16)	0.69	Campaign title sentiment [n]	0.74 (0.35-1.55)	0.43
Infectious disease (ICD-10 A00-B99) n(%)	2.41 (0.51-11.36)	0.27			

CI – Confidence Interval; EUR – Euro; OR – Odds Ratio.
Variables with p-value <0.1 were bolded.

Supplementary File 10

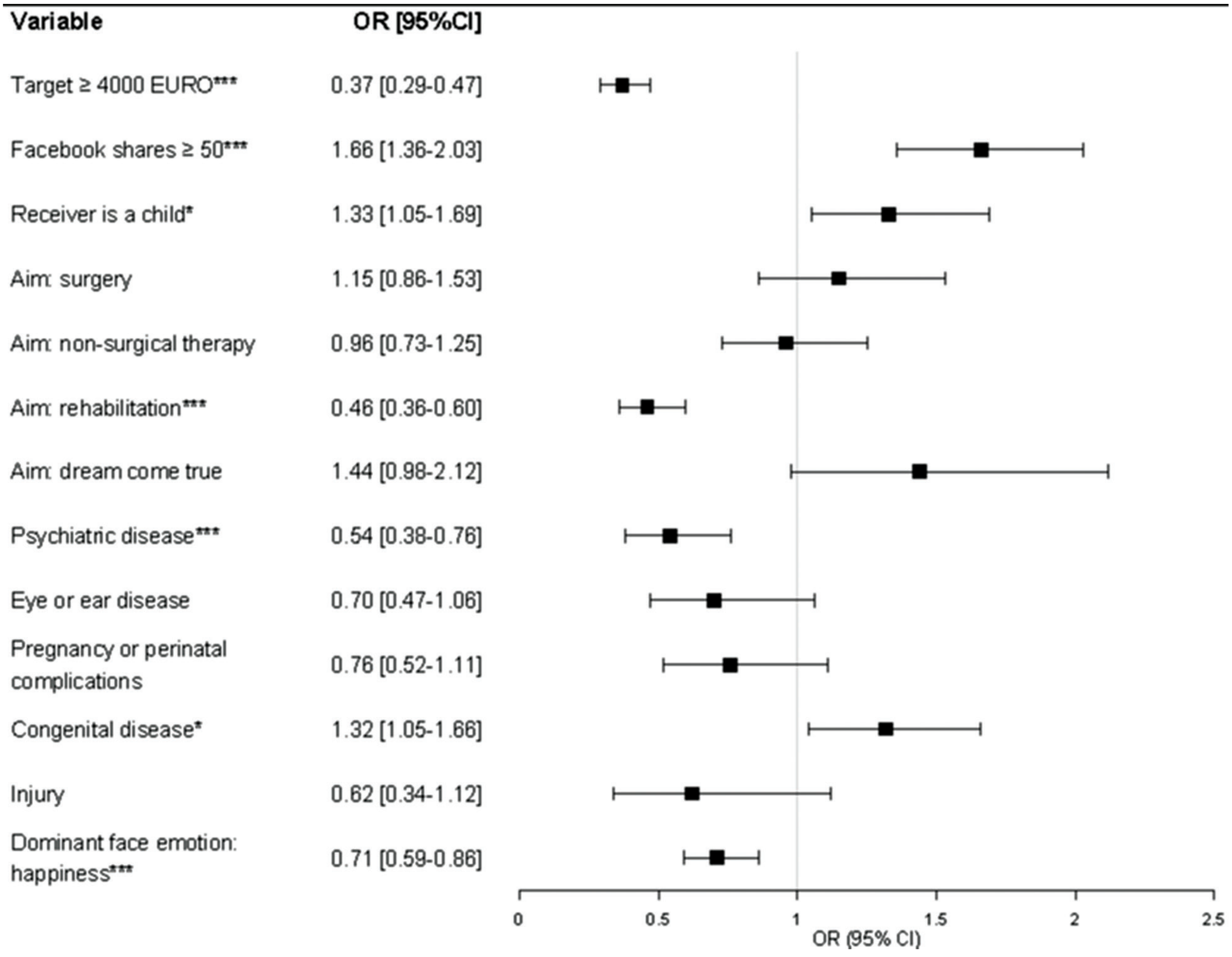

Figure S10. Multivariate regression analysis for campaigns with main photograph face expression analysis (n = 2129). Dependent variable: success of the campaign.
*p < 0.05; **p < 0.01; ***p <0.001.
EUR - Euro, CI - Confidence interval, OR - Odds ratio.

Supplementary File 11

Multiple linear regression analysis.

	β [EUR]	Standard Error	t value	p-value
Number of Facebook shares	5.8	0.2	33.8	< 0.001
Age: Children	9,375.9	2,713.8	3.5	< 0.001
Aim: Surgery	13,951.8	2,749.3	5.0	< 0.001
Aim: Non-surgical therapy	16,943.6	2,917.0	5.8	< 0.001
Intercept	- 9,959.5	2,638.5	- 3.8	< 0.001

Dependent variable: collected sum [EUR]. (adjusted R^2=0.33, F(4,2651)=334.3, p < 0.001).

	β [%]	Standard Error	t value	p-value
Number of Facebook shares	0.0006	0.0001	6.86	< 0.001
Age: Children	7.46	1.53	4.87	< 0.001
Aim: Rehabilitation	-6.71	1.57	-4.27	< 0.001
Aim: “Dream come true”	9.20	2.13	4.32	< 0.001
Psychiatric disease (ICD10 F01-F99)	-6.13	2.43	-2.52	0.01
Eye or ear disease (ICD10 H00-H59)	-7.19	2.96	-2.43	0.01
Intercept	7.44	1.41	52.98	< 0.001

• Dependent variable: percentage of raised target of the collection [%]. (adjusted R^2=0.05, F(6,2649)=23.61, p < 0.001).
